# miRquad: first-in-class dPCR multiplex TaqMan™ Advanced clinical research assay for microRNA detection in head and neck cancer

**DOI:** 10.1186/s13046-025-03590-6

**Published:** 2025-12-20

**Authors:** Matteo Allegretti, David J. Joun, Giulia Urbani, Valentina De Pascale, Federica Ganci, Raul Pellini, Giada Anna Beltramini, Stefano Ferrero, Stefano Fiori, Tania Moccia, Chiara Ciardiello, Elena Di Gennaro, Alfredo Budillon, Luca Sigalotti, Roberta Maestro, Mario Urtis, Eloisa Arbustini, Simona De Summa, Amalia Azzariti, Stella Gagliardi, Antonio Pisani, Gennaro Ciliberto, Paola Cornelia Maria Muti, Junko F. Stevens, Giovanni Blandino

**Affiliations:** 1 Translational Oncology Research Unit, IRCSS Regina Elena National Cancer Institute, Rome, Italy; 2 Thermo Fisher Scientific, Carlsbad, USA; 3 Otolaryngology - Head and Neck Surgery, IRCCS Regina Elena National Cancer Institute, Rome, Italy; 4 Division of Pathology, Fondazione IRCCS Cà Granda, Ospedale Maggiore Policlinico, Milan, Italy; 5 Department of Biomedical Surgical and Dental Sciences, University of Milan, Milan, Italy; 6 Experimental Pharmacology Unit, Istituto Nazionale Tumori – IRCCS Fondazione G. Pascale, Naples, Italy; 7 Neoplastic Progression Unit, Istituto Nazionale Tumori – IRCCS Fondazione G. Pascale, Naples, Italy; 8 Scientific Directorate, Istituto Nazionale Tumori – IRCCS Fondazione G. Pascale, Naples, Italy; 9 Oncogenetics and Functional Oncogenomics, Centro Di Riferimento Oncologico (CRO) IRCCS, Aviano, Italy; 10 Centre for Inherited Cardiovascular Diseases, Fondazione IRCCS Policlinico San Matteo, Pavia, Italy; 11 Experimental Pharmacology Laboratory, IRCCS Istituto Tumori Giovanni Paolo II, Bari, Italy; 12 IRCCS Mondino Foundation, Pavia, Italy; 13 Brain and Behavioral Sciences, University of Pavia, Pavia, Italy; 14 Scientific Directorate, IRCCS Regina Elena National Cancer Institute, Rome, Italy

**Keywords:** Digital PCR, Multiplex assay, MicroRNA, Head and neck cancers, Liquid biopsy

## Abstract

**Background:**

Cancer resistance is one of the major challenges in oncology, often resulting in disease relapse and poor patient outcomes. Within the RNA family, microRNAs (miRNAs) regulate core biological processes and have been recognized also as critical contributors of tumor resistance and therapy failure. Being pivotal, they are increasingly exploited as biomarkers in various settings. Although in silico analyses facilitate miRNAs identification, PCR-based approaches remain essential to validate their expression. Currently, a plethora of well-established, single-target methods exist but multiplex detection from the same input have been only rarely explored.

**Methods:**

We present miRquad, the first-in-class digital PCR (dPCR) TaqMan™ multiplex clinical research assay for miRNA detection in head and neck (HNC) cancers. Based on a patented prognostic signature including miR-21-5p, miR-96-5p, miR-21-3p and miR-429, the assay would enable simultaneous miRNA analysis via qPCR and dPCR on multiple clinically relevant sample types.

**Results:**

We designed and optimized miRquad using both synthetic controls and retrospective patient-derived tissues, sera and saliva. A multicentre ring study was conducted to evaluate assay reliability across different platforms, demonstrating strong correlation with commercial singleplexes, broad applicability, reduced turnaround time (TAT) and cost-effectiveness. Finally, we provide evidence for its potential clinical application to predict disease outcome in HNC, testing miRquad on tumoral and peritumoral tissues, sera and saliva samples collected throughout patient follow up.

**Conclusions:**

The assay overcomes common challenges associated with multiple miRNAs detection, particularly in liquid biopsy samples (e.g., multiple pipetting issues, increased consumption of sample for multiple assessment, extended TAT for complete profiling) and provides robust and accurate detection, demonstrating potential for real-time patient monitoring and prognostication in HNC.

**Supplementary Information:**

The online version contains supplementary material available at 10.1186/s13046-025-03590-6.

## Background

Despite remarkable therapeutic achievements, cancer resistance remains a key hurdle in oncology, often causing poor drug responses, disease relapse and, ultimately, patient death [1]. Tumors escape treatment pressure through multiple mechanisms [2], also including non-genetic alterations [3]. Although our understanding has considerably expanded over the years, tools to anticipate tumor evolution or predict therapy failure remain limited, leaving cancer resistance a pressing challenge in the era of precision medicine.

MicroRNAs (miRNAs) are a class of single-stranded, small non-coding RNA molecules that post-transcriptionally modulate gene expression [4, 5]. Due to their pivotal role in various physiological and pathological processes, including cancer, miRNAs have attracted increasing attention as diagnostic and prognostic biomarkers, being also investigated for therapeutic purposes both in tissues and liquid biopsies (e.g., circulating miRNAs) [6–8]. Moreover, their function as key determinants of cancer resistance and drug metabolism has been elucidated [9]. However, intrinsic features such as their size, variable abundance in body fluids, sequence homology within different families combined with technical hurdles (e.g., inconsistencies across extraction methods, multiplexing requirements, lack of standardized strategies for normalization of target expression) still pose challenges for miRNA identification and clinical exploitation, limiting their stable implementation as cancer biomarkers [10, 11].

So far, numerous approaches to evaluate miRNA expression levels, alone or as components of broader signatures, have been proposed [11, 12]. Among them, reverse transcription quantitative PCR (RT-qPCR) is widely recognized as the gold standard, showing enough sensitivity, specificity and a broad dynamic range while performing at relatively low costs in different settings [13, 14]. However, the investigation of single deregulated miRNAs, typical of standard RT-qPCR workflows, is often inadequate for cancer profiling, which requires quantitative and multiplex analyses [15]. RT-qPCR also faces limitations when applied to challenging sample types, like those characterized by a limited number of analytes (e.g., liquid biopsies) or by the presence of polymerase inhibitors. Additionally, screening large collections of samples looking for even moderate numbers of targets is prohibitive with qRT-PCR and singleplex assays, requiring multiple individual reactions per biomarker. This, in turn, may result in an error-prone and inefficient process due to sample splitting and wasting [14, 16]. Over the years, alternative methods as microarray or NGS sequencing have been introduced providing larger amounts of data but in a relatively slow and expensive fashion [17, 18]. Drawbacks have emerged also for other techniques as rolling circle amplification, duplex-specific nuclease signal amplification, isothermal amplification, all demanding for reaction optimization, improvements in signal readouts, reproducibility and accuracy [19–21]. Besides, the introduction and constant update of novel digital PCR (dPCR) systems, either in droplet or array-based format, are expanding the possibilities for accurate miRNAs profiling, particularly in liquid biopsy applications, paving the way for transforming these analytes in powerful, non-invasive tools for cancer detection and monitoring [22, 23]. dPCR offers absolute quantification of targets by partitioning reactions into thousands of droplets or wells and applying Poisson statistics [24]. This approach reduces the need for external calibrators or normalization controls, enhances analytical sensitivity, and reduces hands-on time compared to conventional methods. Moreover, advanced platforms support multiplexing as compared to 1st-gen dPCRs [25] enabling cancer-related miRNA signatures to be explored with reduced workload, input requirements and at sustainable costs. Nonetheless, challenges related to probe cross-reactivity and assay design may arise, requiring efforts before introducing multiplexed miRNA-based liquid biopsy analyses in clinical practice.

Here, we present miRquad, the first-in-class dPCR TaqMan™ Advanced clinical research assay for multiplex miRNA detection in head and neck (HNC) cancers. Based on our previous works demonstrating the prognostic value of miR-21-5p, miR-96-5p, miR-21-3p and miR-429 signature in predicting HNC local recurrence [26–28], we designed and validated a custom research assay relying on TaqMan™ Advanced chemistry, benchmarking its performance on different cohort of clinical samples (e.g., FFPEs, sera and saliva) and lab settings (i.e., through a dedicated ring study). Our efforts result in reproducible, accurate, and affordable quantification of target miRNAs, comparable to that of TaqMan™ commercial single research assays, but with significant advantages over conventional investigation such as lower sample consumption, limited pipetting issues, multiplexing capacity, reduced TAT, increased efficiency and scalability. Overall, these findings support the potential translation of miRquad into routine clinical practice for the management and monitoring of HNC.

## Methods

### Custom miRNA mimics and spike-ins development

To mimic the presence of target miRNAs in solution and dispose of soluble ‘standards’ for a flexible investigation, both in terms of concentration (relative abundance) and analytical composition (numbers of miRNAs simultaneously present), without facing with the biological complexity of clinical samples, 4 different custom BLOCK-IT™ RNA (Thermo Fisher Scientific, Carlsbad, CA, USA) oligonucleotides were designed using the online tool BLOCK-iT™ RNAi Designer (https://rnaidesigner.thermofisher.com/rnaiexpress, Thermo Fisher Scientific) based on the reported sequences in the miRBase database (release 22, https://www.mirbase.org) (Supplementary Table 1). These products were chemically modified at 5’ with the addition of a phosphate group, and at 3’ to show protruding ends. Both modifications were necessary to allow reverse transcription as for endogenous miRNAs. Custom BLOCK-IT™ RNAs were resuspended in nuclease-free water at a fixed concentration (100 µM) and subsequently diluted at scalar doses (dilution factor 1:10) to generate (a) different single spike-ins hosting known concentrations of a single miRNA of interest and (b) a multiple spike-in that simultaneously included all 4 different miRNAs at 4 different concentrations, which were chosen based on our previous studies [26, 27] (Supplementary Table 2). Synthetic spike-ins were then stored at −80°C until RNA extraction.

### Biological materials

Formalin-fixed paraffin-embedded tissue samples (FFPE, *n* = 60), including primary tumors and matched peritumoral area from treatment-naïve HNC patients, were obtained from Policlinico of Milan based on an ongoing collaboration within a national grant (PNRR-POC-2022–12376580, EC authorization #105/IRE/24) and used as a validation cohort. Additional FFPE samples (*n* = 24), belonging to newly-diagnosed HNCs with different clinical outcome and no history of previous treatment, were obtained from the Regina Elena National Cancer Institute biobank (BB-IRE) and applied for the preliminary benchmark of miRquad and the ring study (see below). FFPE Sects. (5 µm) were initially assessed with immunohistochemistry for tumor content by a dedicated pathologist and further used for nucleic acid extraction as described below. Serial liquid biopsy samples including sera (*n* = 48) and saliva (*n* = 16), collected at specific time intervals (i.e., pre-surgery, 1 day post-surgery, 15 days post-surgery, and at follow-up visits), were retrospectively obtained from the BB-IRE. These were part of a prospective study underway at IRE (EC authorization #RS868/16) which involves patients affected by HPV-negative, resectable HNC of the oral cavity, pharynx or larynx undergoing surgery with curative intent and chemo/radio-treatments or in follow-up as per clinical practice. Study cohort was selected based on the availability of the clinical outcome (i.e., patients with favourable or unfavourable prognosis) and a minimum follow-up of 36 months. Exclusion criteria were as follows: patients with previous (before surgery) and/or ongoing chemo/radiotherapy, metastatic disease, history of previous (< 5 years before enrolment) or concomitant tumor(s) of different histology. A written informed consent was collected from all subjects involved in the study. With respect to sample nature, biological fluids have been subjected to single centrifugation at 3500 g for 20 min (sera) or at 2000 g for 10 min (cleared saliva), both at 4 °C. The former were stored at −80 °C while the latter were kept at room temperature by adding 10 ml of ThinPrep solution (Hologic BV, Zaventem, Belgium) to allow long-term stabilization. A complete list of biological samples is presented in Supplementary Table 2.

### miRNAs extraction from synthetic and biological specimens

Extraction of total RNAs, including miRNAs of interest, from synthetic materials, tissues and liquid biopsies was carried out by the MagMAX™ mirVana™ Total RNA isolation kit and the KingFisher™ Apex purification system (both from Thermo Fisher Scientific), according to the manufacturer instructions, in a final volume of 50 µl (tissues) or 30 µl (spike-ins, liquid biopsies), respectively. Ten nanograms of Qubit™ BR (Thermo Fisher Scientific) quantified RNAs from tissues or 2 µl of eluate from liquid biopsies were then reverse transcribed using the TaqMan™ Advanced miRNA cDNA synthesis kit (Thermo Fisher Scientific), as per protocol. This supports optimal conversion of miRNAs in the corresponding cDNA, avoiding the need for specific oligonucleotides and targeted amplification, thus providing a single product including all targets at the original ratios. Resulting cDNAs were stored at −20 °C until downstream amplification by qPCR or dPCR.

### qPCR and dPCR analyses

Analysis of miRNA expression (i.e., miR-21-5p, miR-96-5p, miR-21-3p and miR-429) was carried out by using either commercially available singleplex TaqMan™ Advanced research assays, single custom assays (those composing the miRquad) or the miRquad as a whole on the QuantStudio™ 5 real-time PCR and QuantStudio™ Absolute Q digital PCR systems (both from Thermo Fisher Scientific). Experimental design aimed at providing (a) pre-validated commercial references, (b) evaluating the performance of the multiplex assay and (c) investigating any quantitative issue or fluorescence interference due to the simultaneous amplification and detection of different molecular targets into the same reaction. As for qPCR, reactions were setup in triplicates in a final volume of 20 µl containing 10 µl of 2 × TaqMan™ Advanced master mix, 1 µl of 20 × TaqMan™ research assays (both from Thermo Fisher Scientific), 5 µl of a 1:10 diluted sample and nuclease-free water. Thermocycling conditions were as follows: 95 °C for 20 s, 40 cycles at 95 °C for 1 s and 60 °C for 20 s. miRNAs levels, as assessed by commercial assays, were evaluated through automatic thresholding and the average Ct mean, and internally compared with those of custom assays, either in single or multiplex (miRquad) format. dPCR reactions were prepared in a final volume of 10 µl containing 2 µl of 5 × Absolute Q™ universal DNA master mix, 0.5 µl of 20 × TaqMan™ research assays (both from Thermo Fisher Scientific), 5 µl of 1:10 diluted sample and nuclease-free water. Thermocycling protocol was as follows: 96 °C for 10 min, 40 cycles at 96 °C for 5 s and 60 °C for 15 s. Automatic thresholding was applied by the software (QuantStudio™ Absolute Q dPCR software, v. 6.3.5, Thermo Fisher Scientific), manually reviewed by the operator to enhance discrimination between negative from positive dots, and miRNA levels expressed as copies/µl of reaction. dPCR results were compared across various assay conditions and correlated with qPCR data.

### INNOVA Consortium ring study

To extend the analytical evaluation of miRquad on different qPCR/dPCR platforms and clinical environments, the INNOVA Consortium, a multidisciplinary diagnostic network operating within the Italian NHS and translational research which promotes the development and dissemination of novel, standardized, advanced molecular tools, was considered. A ring study involving various (*n* = 6) Italian Cancer Centres belonging to the WP4 – Liquid Biopsy & Biomarkers group (Supplementary Fig. 1) was designed and executed as follows: synthetic materials (e.g., single and multiple spike-ins) and representative FFPE, sera and saliva samples (*n* = 7, 4 and 4, respectively) available at IRE (Supplementary Table 2), were extracted, reverse transcribed, splitted in single use aliquots and shipped to all participants; receiving lab personnel were invited to analyse them in a blinded manner according to their own laboratory practice, simultaneously applying both the commercial TaqMan™ Advanced miRNA research assays, custom singleplexes composing the miRquad and the miRquad in the same qPCR or dPCR reaction (depending on instrument availability); raw data were preliminary analysed as per laboratory routine using the different on board softwares (depending on the instrument type), afterwards sent to IRE for a more broader and cumulative evaluation.

### Statistical analyses

Evaluation of miRquad performance as compared to commercial assays was performed using Student’s t-test and linear regression. The non-parametric Mann–Whitney test was applied to investigate statistical significance of miRNA changes in patient-derived materials. Wilcoxon matched-pairs signed-rank test was applied for comparisons between paired measures. *P* values ​​ < 0.05 were considered statistically significant. All data were reported using GraphPAD® Prism v. 10.5 software application (GraphPAD® software, Dotmatics).

## Results

### Custom TaqMan™ advanced singleplexes specifically amplify their targets mirroring the performance of commercial research assays

**Fig. 1:**
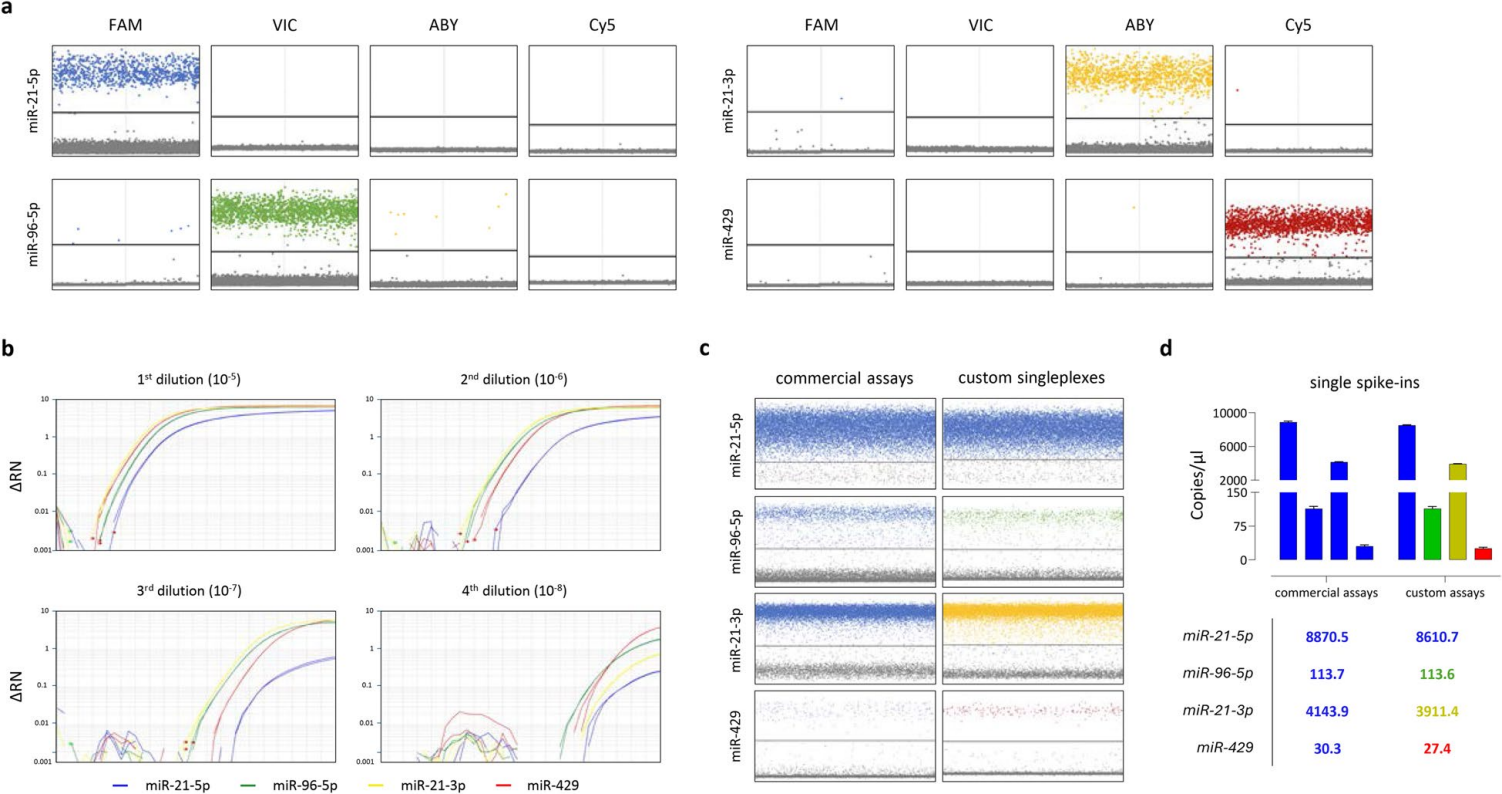
Preliminary analysis of custom assays performances as singleplex. Single spike-ins containing a discrete concentration (⁓100 copies/µL) of each miRNA of interest were used to preliminarily assess amplification, specificity and selectivity of the corresponding custom TaqMan™ Advanced research assay. **a** 2D plots describing the resulting signals obtained from each singleplex, in duplicates, when tested in dPCR. None to negligible spillovers were detected in surrounding channels (indicated). **b** Dilution series of single spike-ins containing scalar concentrations of each BLOCK-iT™ RNA mimicking the miRNA of interest (miR-21-5p, blue; miR-96-5p, green; miR-21-3p, yellow; miR-429, red) were generated and further tested in qPCR using commercial TaqMan™ Advanced assays to evaluate the presence of each target over a wide dynamic range. **c**-**d** Side-by-side comparison of miRNA expression in single spike-ins by either commercial assays (left) or custom singleplexes (right). Mean values and standard deviation (error bars) from *n* = 2 technical replicates are shown. Detection channels are color-coded. Resulting signals appear equivalent in terms of copies/µl of reaction, demonstrating robustness in amplification and quantification of the molecular targets

The formulation of a multiplex assay requires switching from FAM™-conjugated TaqMan™ probes to other fluorescent molecules (e.g., VIC™, ABY™ and Cy5™) to allow simultaneous discrimination of several miRNAs across different channels. To verify whether these modifications may affect the ability of each assay, as singleplex, to properly interact with its target and/or generate false positive spillover-dependent signals (i.e., non-specific signal in neighbouring fluorescence channels), preliminary experiments were performed in qPCR (data not shown) and dPCR using spike-ins containing a discrete concentration (⁓100 copies/µl) of each miRNA of interest as input (Fig. 1). Our results clearly showed the ability of the custom assays, with respect to their own fluorescence channel, to successfully amplify target sequences with elevated selectivity, as demonstrated by the negligible residual signals (spillover) in the non-target fluorescence channels (Fig. 1a). Next, since the different brightness of custom fluorophores as compared to FAM™ may also affect instrument thresholding impairing the ability to properly discriminate positive/negative signals, we assessed whether our custom singleplexes incorrectly quantified the concentration of target miRNAs and if their detection was allowed within a dynamic range sufficiently wide to consider different biological sources. Initially, we generated series of single spike-ins by sequentially diluting each specific BLOCK-IT™ RNAs at a fixed ratio (1:10) and tested them on qPCR by using commercial assays (Fig. 1b). A good intra- and inter-assay correlation in terms of scalar concentration was found. Based on the expected expression of each miRNA in clinical samples, specific dilutions from each spike-in series were selected as follows: miR-21-5p > miR-21-3p > miR-96-5p > miR-429. dPCR experiments were then performed investigating miRNA expression with commercial assays or by applying our custom singleplexes. The analysis of copies/µl ​​demonstrated a significant (*p* < 0.001) concordance ​​between the two detection methods (Fig. 1c-d). Furthermore, no lack of performance was noted with respect to the different concentrations of the BLOCK-IT™ RNA used as input, demonstrating the high sensitivity of the custom products.

### miRquad multiplex shows comparable performance to those of custom TaqMan™ Advanced single research assays

**Fig. 2:**
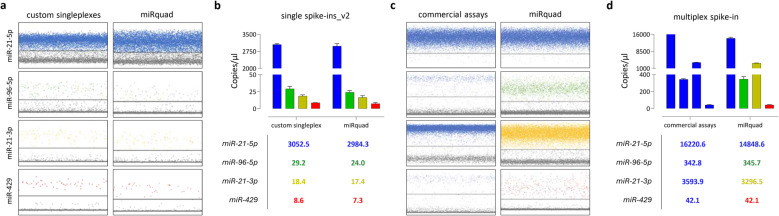
dPCR analysis of miRquad as compared to custom singleplexes on synthetic spike-ins. Single spike-ins containing scalar concentration for each miRNA of interest, previously analysed by qPCR, were used as input to assess the detection capabilities of miRquad. **a** Representative 2D plots depicting miRNA analysis carried out by using either the custom TaqMan™ Advanced research assays as singleplex (left) or the miRquad (right). **b** Mean absolute quantification of copies/µl of reaction (technical replicates = 2) resulting from side-by-side comparison of custom TaqMan™ advanced (left) and miRquad (right) with respect to each miRNA. Detection channel are color-coded. **c** miRquad dPCR testing on the multiplex spike-in, including each single miRNA of interest at decreasing scalar concentrations. **d** Side-by-side comparison of commercial TaqMan™ assays (left) and the miRquad (right) in the multiplex spike-in. Mean absolute quantification of copies/µl of reaction (technical replicates = 2) with respect to each miRNA is shown and color-coded

Having demonstrated the ability of custom assays, as singleplexes, to mirror the behaviour of FAM™-conjugated counterparts, we moved to assess the performance of miRquad as a whole (i.e., in the multiplex format) (Fig. 2). Firstly, a side-by-side comparison was carried out in dPCR on newly-generated single spike-ins by alternatively applying either individual custom assays or the miRquad, aiming at unveiling possible technical issues related to the simultaneous presence of multiple primers and probes in samples including only the target sequence (i.e., the specific miRNA of interest at relatively low abundance instead of a mixture of different miRNAs). We observed that, even in the multiplex configuration, our custom assays retained the ability to discriminate each target. More broader extensions of the positive clusters along the fluorescence axis and a global reduction in fluorescence intensity were noted in almost all of fluorescence channels (Fig. 2a) and, consequently, adjustments of thresholds to ensure correct targets quantification were applied. This effect, partly expected, was supposed to result from the interactions between various primers, their relative concentrations, and multi-probe interferences in the assay. Nevertheless, it did not preclude the use of miRquad nor an accurate quantitation of miRNAs even in the range of low copies/µl (Fig. 2b). To better investigate whether the fluorescence smear was dependent to the formulation of miRquad itself and could increase in samples simultaneously hosting, among different circulating analytes, multiple miRNAs at higher concentrations, becoming detrimental for an accurate quantification, a multiplex spike-in was generated. Different BLOCK-iT RNAs were spiked at decreasing scalar concentrations in nuclease-free water, total RNA extracted, reverse transcribed and cDNAs were used as input for dPCR (Fig. 2c-d). Overall, our data demonstrated that, despite fluorescence smears in all other than FAM™ channel, miRquad supports a consistent and absolute quantitation relative to each specific target of interest, also in the presence of multiple analytes, requiring only minimal adjustments of fluorescence thresholds.

### miRquad is applicable on different biological matrices

**Fig. 3:**
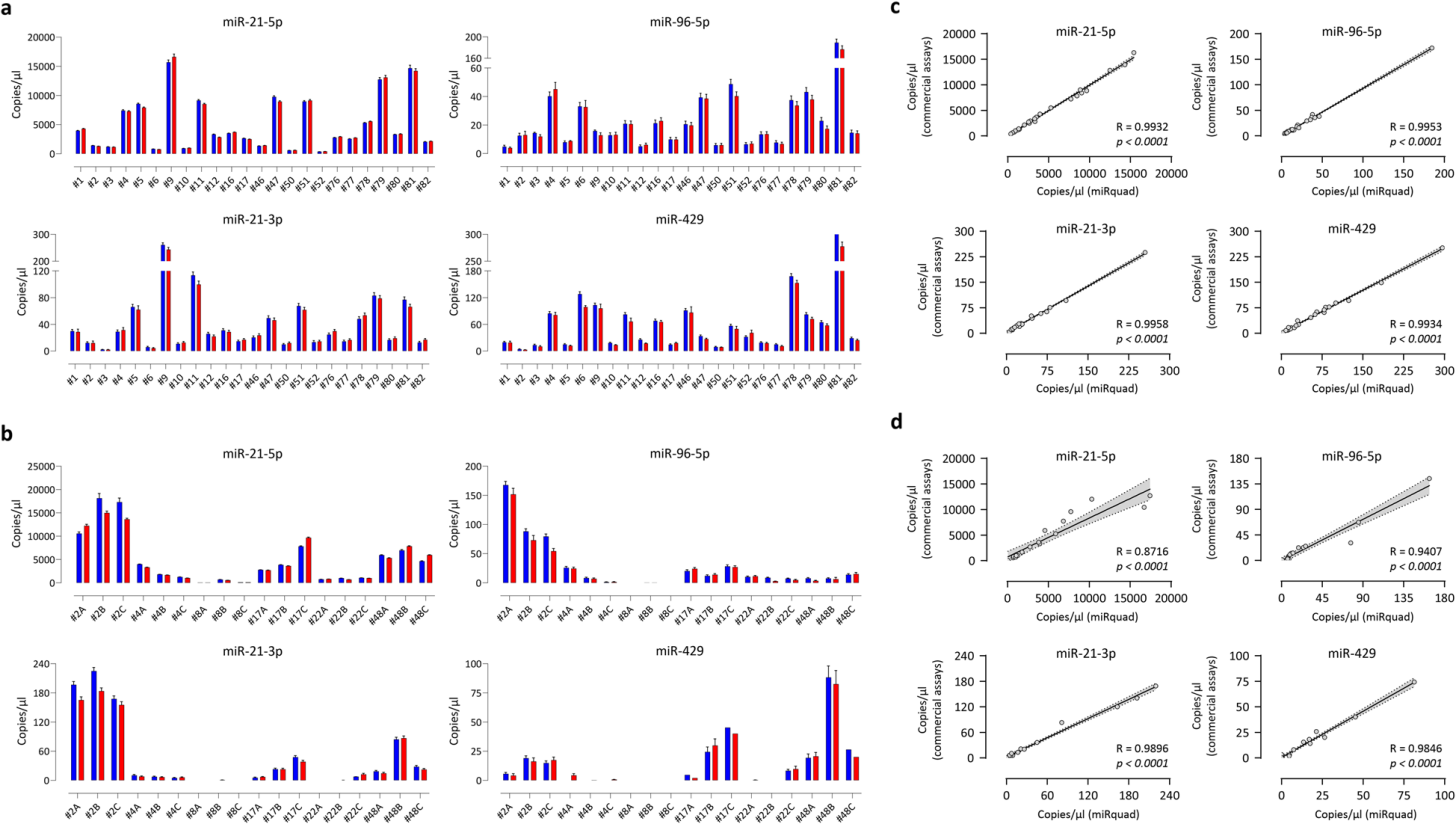
Evaluation of miRquad performances on clinical specimens. Different biological matrices, including FFPE tissues (*n* = 24) and sera (*n* = 18), belonging to HNC patients retrospectively collected at IRE were simultaneously assessed by commercial assays and the miRquad in dPCR. **a**-**b** Analytical comparison of miRNA expression levels in tissue (**a**) and sera (**b**) as detected by commercial assays (blue bars) or miRquad (red bars). Copies/µl of reaction and dPCR precision (error bars) are shown. (**c**-**d**) Linear regression analyses for each specific miRNA of interest in tissue (**c**) or sera (**d**). R and *p* values are indicated

To assess whether miRquad may serve as a valuable molecular tool, particularly in the context of HNC where we previously demonstrated a prognostic role of the signature to predict disease local recurrence [26–28], different biological matrices (i.e., FFPE tissues and sera) were retrospectively collected from the BB-IRE and subjected to dPCR analysis with either the multiplex and commercial assays, the latter being used as a reference. This representative cohort was defined by selecting samples collected over a period of ⁓7 years (range: 3–10 years ago) aiming at mimicking technical and biological variability which generally occurs in real-world clinical settings. Analytical comparisons of assay performances in FFPEs and sera are presented in Fig. 3a-b and Suppl. Tab. 3, respectively. As shown, even where RNAs can be notoriously degraded due to sample processing or quantities of circulating miRNA are expected to be intrinsically variable, miRquad maintained the ability to properly identify each target of interest, as the validated commercial assays, showing a significant (*p* < 0.0001) correlation in terms of copies/µl of reaction across all different tested sources, for all miRNAs (Fig. 3c-d).

### miRquad grant reproducible results over different platforms and clinical contexts

**Fig. 4:**
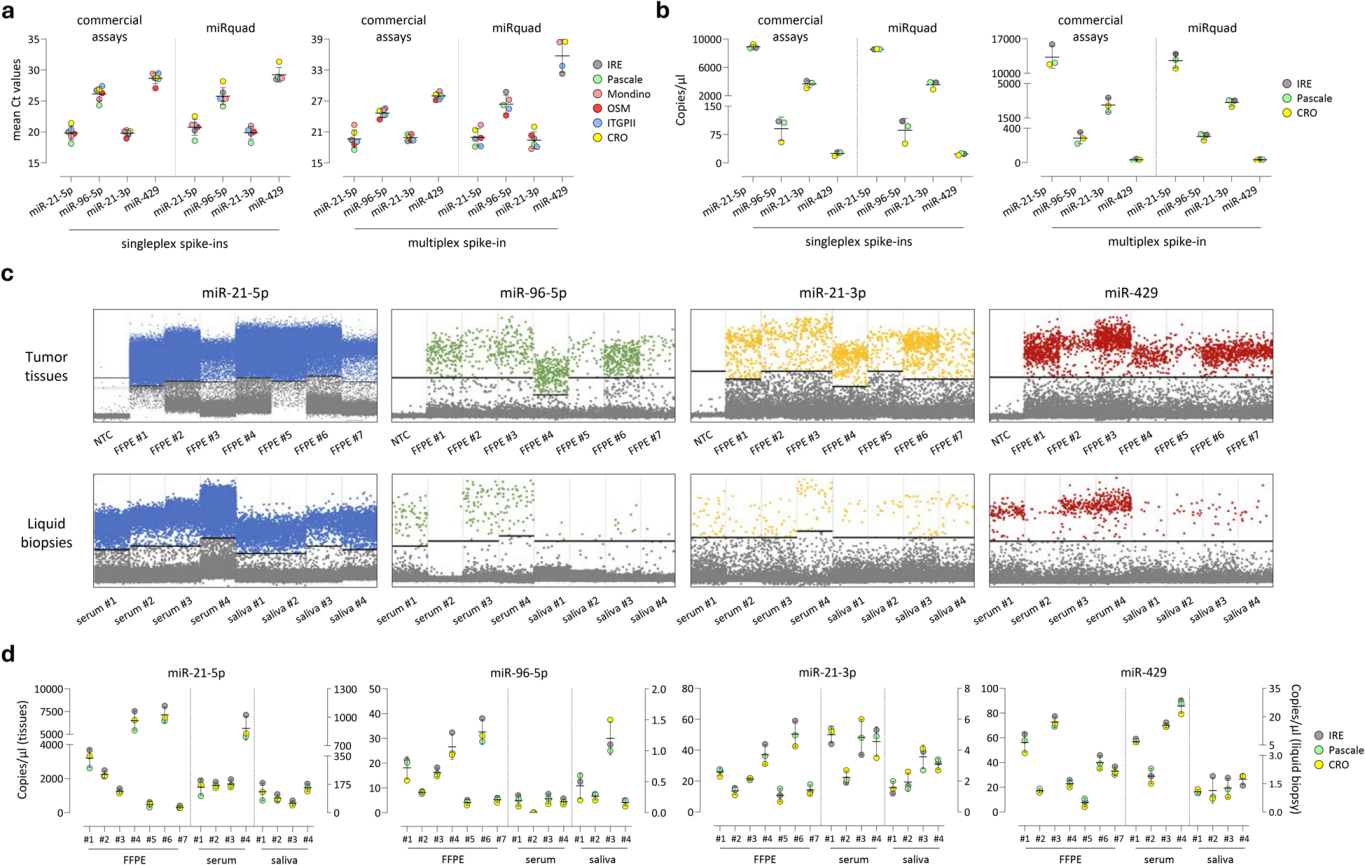
Extended analysis of miRquad across different Italian cancer Centers. A ring study involving 6 Italian Institutes belonging to the INNOVA Consortium and the WP4 – Liquid Biopsy group was executed to benchmark miRquad robustness on different qPCR/dPCR platforms and in the context of variable technical expertise. **a**-**b** Analysis of miRNA expression levels detected by commercial assays (left) or the miRquad (right) in single and multiplex spike-in products by qPCR (a) or dPCR (b). Values obtained in each specific Center are indicated by color-coded dots. **c** Representative 2D plots showing miRNA abundance in patient-derived materials included into the ring study as analyzed at IRE by dPCR. **d** Multicenter comparison of miRNA expression levels in tissues and liquid biopsies by dPCR. Copies/µl of reaction are shown (tissue: left axis; sera and saliva: right axis). NTC: no template control (nuclease-free water)

To corroborate preliminary findings, and technically benchmark assay performances on different qPCR/dPCR platforms and in contexts featured by variable technical expertise, evaluation of miRquad was extended by performing a ring study involving 6 Italian institutes belonging to the INNOVA Consortium (Supplementary Fig. 1). Briefly, cDNAs resulting from synthetic material (i.e., spike-ins, both as single or multiplex) and the above-cited biological specimens were shipped in ready-to-use aliquots, blinded to the final users who were asked to provide a final data report according to their laboratory practice. All the results were then collected by IRE, revised to exclude technical and biological issues, and subjected to comparative analysis. As shown in Fig. 4, the application of commercial assays or the miRquad for qPCR (Fig. 4a) or dPCR (Fig. 4b) analyses resulted in a minimal (mean qPCR CV = 6.2%; mean dPCR CV = 3.5%) variability in terms of mean Ct outputs or copies/µl of reaction, respectively, across the different Centers in both single and multiplex spike ins. Highest CVs were recorded on Cy5 fluorescence (i.e. the expression of miR429). This effect was partially expected due to the lower brightness of the fluorophore which renders signal quantification less efficient as compared to the other dyes. Then, representative patient-derived materials, either FFPEs or liquid biopsies (i.e., sera and saliva), were addressed in dPCR with miRquad (Fig. 4c). Again, no significant differences in miRNA expression levels were noted between different Centers (Fig. 4d). Overall, no technical hurdles were recorded by running miRquad on qPCR/dPCR platforms provided by different vendors, thus supporting its applicability as a molecular tool for biological investigation.

### miRquad as a non-invasive tool for predicting local recurrence risk in HNC

**Fig. 5:**
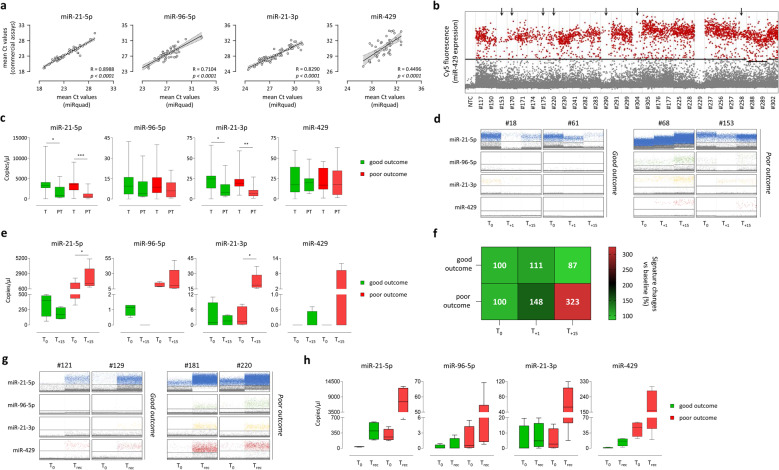
Validation of miRquad on independent cohorts of patient-derived samples. Translational potential of miRquad for HNCs was investigated by qPCR and dPCR on a cohort of independent FFPE samples (*n* = 60), sera (*n* = 48) and saliva (n = 16). **a** Linear regression analyses of mean Ct values obtained by commercial assays or the miRquad in tissue samples for each specific miRNA. R and *p* values are shown. **b** Representative 1D plots of miR-429 expression (Cy5 fluorescence) in low-abundance FFPEs analyzed by miRquad in dPCR. Samples previously missed by qPCR or showing discrepancies between standard (i.e., with commercial assays) and miRquad-based analyses are indicated by the arrows. **c** miRNA expression assessed by dPCR and the miRquad assay in tumor tissues and matched peritumoral area as related to the clinical outcome. **d** Longitudinal evaluation of circulating (sera) miRNA levels in patients with good (left panels) and poor outcome (right panels). Representative 2D plots are shown. T_0_: before surgery; T_+1_: 1 day after surgery; T_+15_: 15 days after surgery. **e** miRNA expression levels, as quantified by dPCR and the miRquad assay, in sera samples of good and poor responders at baseline (T_0_) and 15 days after surgery (T_+15_). **f** Heatmap summarizing signature modulations occurring in good (up) and poor (down) outcome patients in the immediate post-surgery follow up. Values are expressed as percentages of mean changes *vs* baseline. **g** Representative 2D plots of miRNA expression in saliva samples of good (left) and poor (right) outcome patients obtained at baseline (T_0_) and before relapse (T_rec_). **h** Comparative analysis of miRNA expression levels in saliva samples of good (left) and poor (right) outcome patients. * *p* = 0.05; ** *p* < 0.01; *** *p* < 0.001. NTC: no template control (nuclease-free water)

Translational feasibility of miRquad for the clinical management of HNC cancers was finally explored by qPCR and dPCR on an independent cohort of FFPE samples (*n* = 60), including matched tumoral and peritumoral tissues, additional HNC sera (*n* = 48) and a selection of saliva (*n* = 16) (Supplementary Table 2). As previously observed (Fig. 3a), also in these additional tissue specimens no significant differences in the expression levels of miR-21-5p, miR-96-5p and miR-21-3p were observed between the miRquad and commercial assays in qPCR (Fig. 5a). Higher deviations were noted for miR-429 (R^2^ = 0.4496) probably due to the relatively low expression (mean Ct > 30) in these samples and the reduced brightness of Cy5. They were partially mitigated by manual thresholding but, as expected, they completely disappeared in dPCR, which offered a more reliable analysis of miRNA expression, rescuing also those samples originally missed/discordant by qPCR. An extended representation of dPCR analysis in low-abundance samples is shown in Fig. 5b. Notably, when samples were stratified according to sampling site (i.e., core or peritumoral area), miRquad-based analysis recapitulated our previous findings [26, 27], with tumor tissues expressing higher levels of the miRNA signature as compared to the peritumoral area (Fig. 5c). As to liquid biopsies, performances of miRquad were confirmed also in these sera, where dynamic changes occurring during clinical follow-up were easily detected by dPCR (Fig. 5d). Particularly, we demonstrated that HNC patients with unfavourable prognosis were characterized by an increase, easily detectable already 15 days after surgery, of the various miRNAs, particularly those belonging to the miR-21 family, while subjects showing a favourable outcome did not, except for miR-429 (Fig. 5e) whose upregulated levels may depend from its dual role as tumor suppressor/oncomiR [29]. Indeed, as tumor suppressor this miRNA may inhibit epithelial–mesenchymal transition (EMT), metastasis and tumor invasion while, in different contexts (e.g., tumor type, cellular context, and target gene networks), it can promote proliferation, migration, and chemoresistance by suppressing tumor-inhibitory genes [30–32]. However, when signature changes were considered and compared to miRNA baseline levels, the two groups of patients were stratified with higher accuracy (Fig. 5f). Finally, since saliva is recognized as the most suitable source for conducting liquid biopsy analyses in HNC [33, 34], selected samples collected at baseline and before relapse were tested with miRquad. Representative results are shown in Fig. 5g. Also in this context, miRquad-based detection resulted in a clear evaluation of each miRNA of interest, reinforcing the observation of higher signature expression in patients with poor prognosis as compared to those displaying good outcome (Fig. 5h), thus suggesting a potential use of miRquad for real-time monitoring and patient prognostication in HNC by miRNA analysis.

## Discussion

Over the last few years, growing evidence have highlighted miRNAs as promising tools in precision medicine, particularly in oncology. As master regulators of physiological and pathological gene expression, miRNA offer several advantages: (a) broad expression, enabling detection from different sources; (b) high stability in body fluids, allowing non-invasive testing; (c) functional link to key cellular processes underlying disease onset and evolution, and therapy failure [4, 5]. Although ideal as surrogate biomarkers [12, 35], clinical translation of miRNAs is still hindered by technical variability, limited multiplexing capacity, and challenges in achieving inter-laboratory standardization [36]. General guidelines on miRNA handling have been recently released [37] but analytical and post-analytical issues remain less standardized [38–40]. Particularly, conventional RT-qPCR methods, while considered the gold standard for miRNA quantification, typically allow the analysis of only one target per reaction, thereby increasing sample consumption, TAT and analytical variability. High-throughput alternatives such as NGS or microarrays grant broader profiling capabilities but are time-consuming, costly, and less suitable for routine clinical testing. Recently, the introduction of 2nd-gen dPCR has provided solutions to several common issues [22, 25, 41–43] but they are not routinely used for miRNA analysis, and multiplexing capabilities, often critical for oncology applications, are still limited with only a few reports of multiplex assays to date [44–47].

We present miRquad, the first-in-class dPCR TaqMan™ Advanced clinical research assay for multiplex miRNA detection in HNCs. This assay represents a major methodological advance, enabling simultaneous quantification of up to four prognostically relevant miRNAs (i.e., miR-21-5p, miR-96-5p, miR-21-3p, and miR-429) within a single reaction. By doing so, it overcomes key limitations of current singleplex assays, enhancing analytical efficiency, reproducibility, and throughput while reducing sample input and operator-dependent variability. Extensive optimization using single or multiple miRNA spike-ins were performed to reduce potential interference from complex human matrices, both solid (tissues) or liquid (serum, saliva). Consistent with prior recommendations emphasizing adequate miRNA expression levels for reliable detection (⁓100 cp/μL) [48], our custom assays specifically amplified synthetic targets, matching the performance of commercial TaqMan™ RUO assays. Across a broad dynamic range, we observed negligible background, high linearity, and limits of detection comparable to previously reported designs [42–44, 48]. Although multiplexing led to a modest reduction in fluorescence intensity, a predictable and addressable issue through additional fluorophore adjustment, absolute miRquad-driven quantification remained unaffected. Besides technicalities, our assay demonstrates significant clinical potential. Translational feasibility of miRquad was explored by assessing different cohorts of FFPE samples, sera and saliva longitudinally collected from HNC patients with different clinical outcome. In these contexts, miRquad emerged as a natural and effective solution, enabling improved performance (e.g., reduced TAT, costs, sample inputs, and minimizing handling steps) while recapitulating previous findings [26–28]. The ability to monitor dynamic miRNA fluctuations in serial liquid biopsies strongly supports its use as a minimally invasive tool for real-time monitoring of disease evolution and treatment response, complementing existing conventional imaging and histopathological approaches. Also, the benchmarking performed under real-world conditions, i.e. characterized by intrinsic variability in terms of samples and pre-analytical handling, confirmed the robustness of the assay, underscoring its potential to serve as a valuable molecular tool. Assay reproducibility across multiple centers within the INNOVA Consortium demonstrated readiness for standardization and scalability in clinical laboratories. Finally, compared to existing technologies, miRquad bridges the gap between dPCR precision and the multiplexing efficiency needed for a complete miRNA targeted profiling. Unlike conventional singleplex TaqMan™ assays, it enables concurrent detection without sacrificing accuracy, and compared to NGS, it offers a faster, cost-effective, and easily implementable workflow suitable for diagnostic pipelines. Taken together, these findings supports miRquad as a technically robust solution for multiplex miRNA detection, accelerating the translation of circulating miRNAs into reliable biomarkers for precision oncology in HNC. Despite these promising outcomes, some limitations still remain. The real clinical impact of the assay could not be thoroughly evaluated due to the relatively small size of the sample cohort. While this was beyond the scope of the present study, integrating miRquad into clinical practice will likely require validation in larger, prospective cohorts to fully establish its translational impact. Additionally, technical limitations related to the instrument applied for miRNA analysis (only 4 different targets allowed per time) prevented data normalization using endogenous miRNA or global mean strategies [17, 49]. Nevertheless, our assay was designed to be expandable, accommodating additional miRNAs or spike-in controls, thus granting more controlled measurements. Finally, we had chances to test a limited selection of qPCR/dPCR instruments, while broader comparative studies across a variety of platforms would be more beneficial for establishing robustness and versatility.

## Conclusion

miRquad represents a first-in-class digital PCR–based TaqMan™ Advanced multiplex assay for the simultaneous detection of multiple prognostic microRNAs in HNC. By detecting up to four targets in a single reaction, it enhances analytical efficiency, reduces sample consumption and pipetting issues, and enables high reproducibility across different specimen types and platforms. Its ability to accurately monitor dynamic changes in liquid biopsies highlights potential for non-invasive patient monitoring and outcome prediction in HNC. Collectively, our findings positioned miRquad as a pivotal step toward the integration of advanced multiplex microRNA diagnostics into precision oncology.

## Supplementary Information

Supplementary Material 1: Suppl. Tab. 1. miRNA sequences used for custom BLOCK-IT™ RNAs design.

Supplementary Material 2: Suppl. Tab. 2. Complete list of biological materials used into the manuscript, including sample type, ID, source and application.

Supplementary Material 3: Suppl. Tab. 3. miRNA expression levels by commercial assays and the miRquad. Copies/µl for each miRNA target as detected by dPCR and commercial assays (indicated as cmiR) or the miRquad in (a) tissues and (b) sera samples.

Supplementary Material 4: Suppl. Fig. 1. Footprint of Centers participating in the ring study. Centers and platform availability (qPCR or dPCR) involved into the ring study are indicated.

## Data Availability

Raw data and related analyses supporting paper findings are available upon reasonable request to the corresponding author (GB).

## Abbreviations

dPCR: digital PCR
FFPE: Formalin-fixed paraffin-embedded
HNC: Head and neck
miRNAs: microRNAs
RT-qPCR: Reverse transcription quantitative PCR
TAT: Turnaround time
