# Supplementary figures and images for: miRquad: first-in-class dPCR multiplex TaqMan™ Advanced clinical research assay for microRNA detection in head and neck cancer
Source: J Exp Clin Cancer Res. 2025 Dec 20;45:26. doi: 10.1186/s13046-025-03590-6 (PMC12853808; doi:10.1186/s13046-025-03590-6)

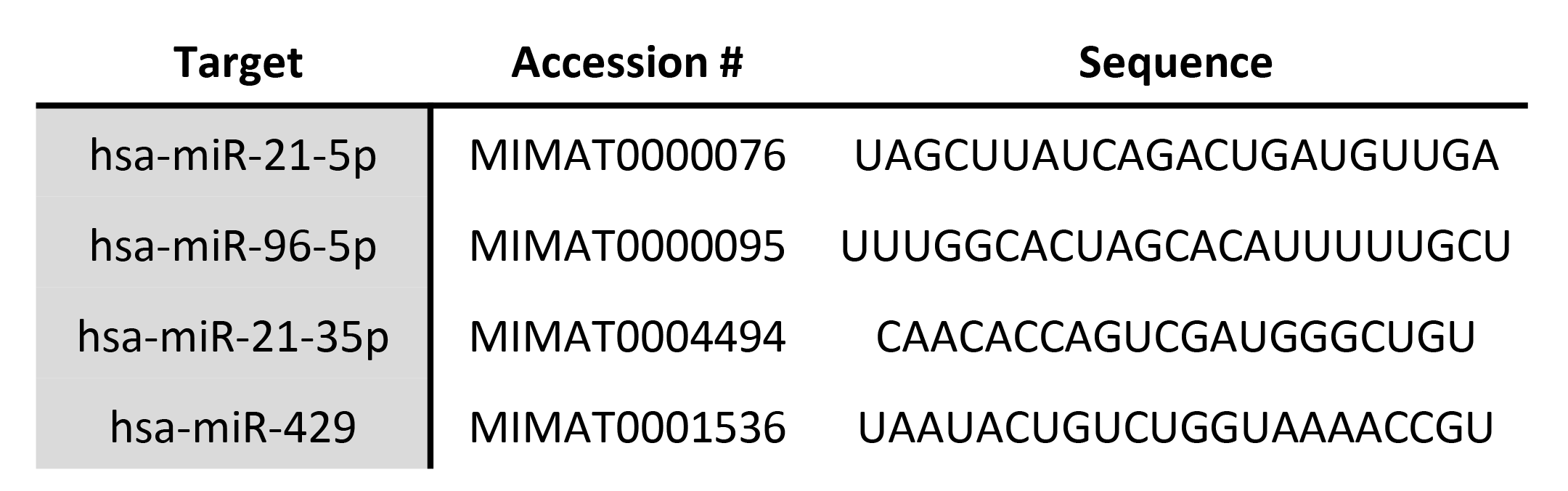

Supplement: Supplementary file 1 — Supplementary Material 1: Suppl. Tab. 1. miRNA sequences used for custom BLOCK-IT™ RNAs design. [file 13046_2025_3590_MOESM1_ESM.tif]

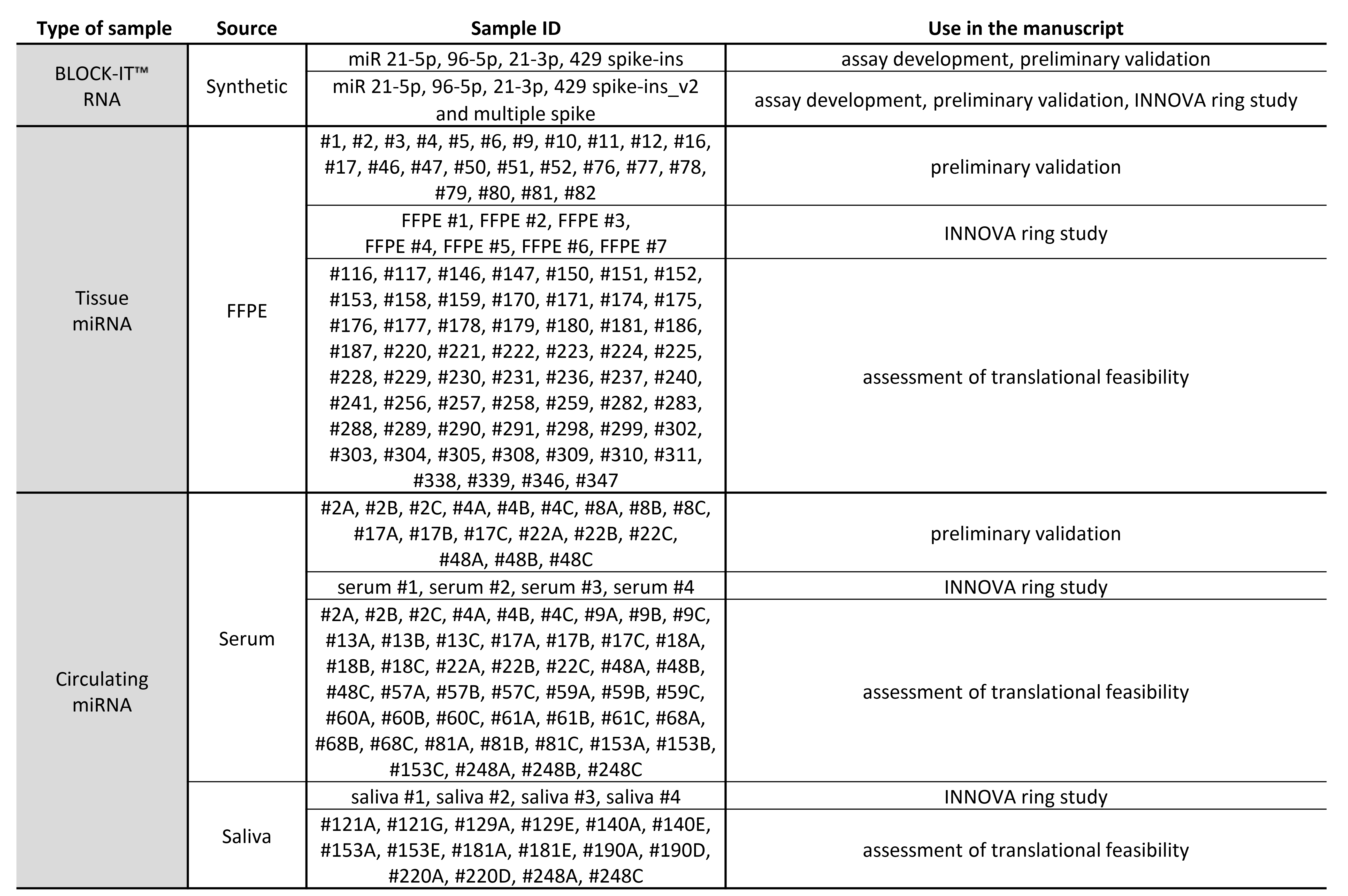

Supplement: Supplementary file 2 — Supplementary Material 2: Suppl. Tab. 2. Complete list of biological materials used into the manuscript, including sample type, ID, source and application. [file 13046_2025_3590_MOESM2_ESM.tif]

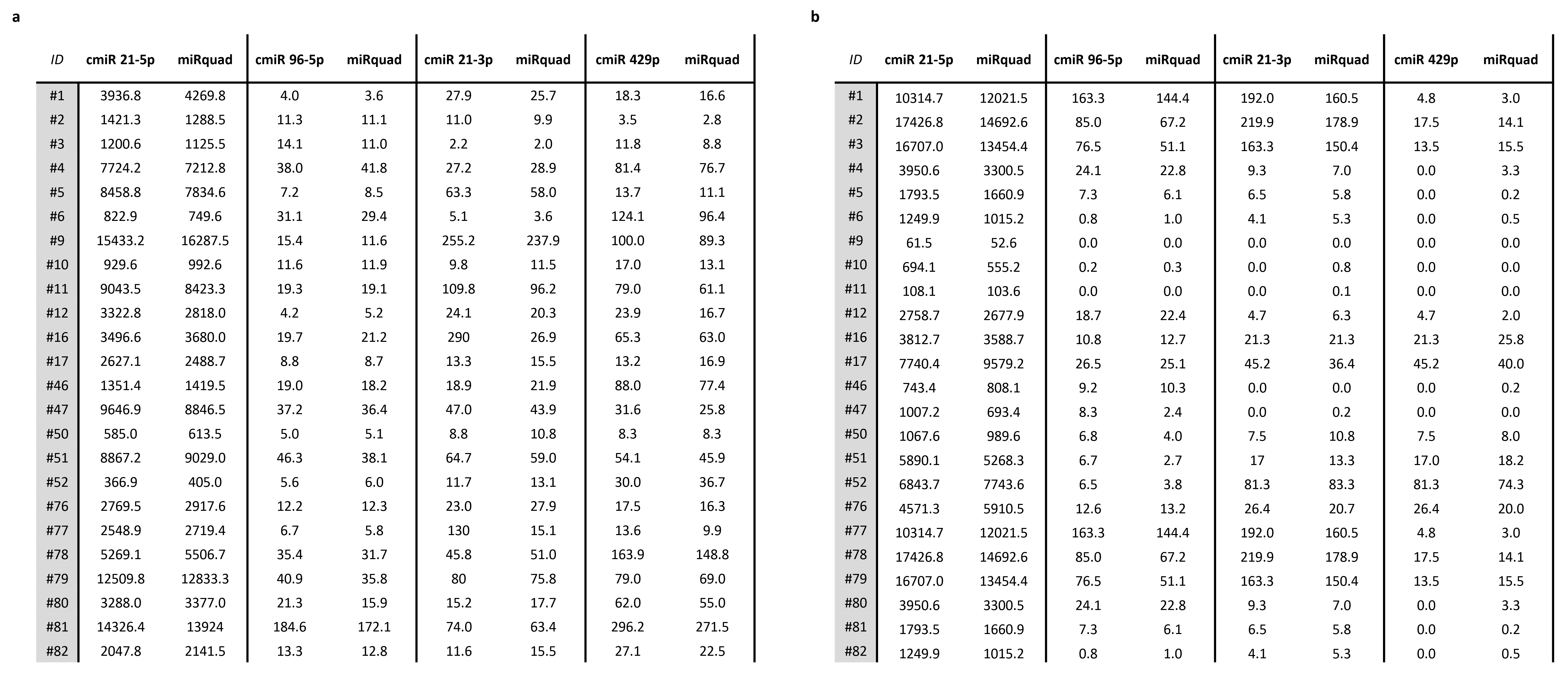

Supplement: Supplementary file 3 — Supplementary Material 3: Suppl. Tab. 3. miRNA expression levels by commercial assays and the miRquad. Copies/µl for each miRNA target as detected by dPCR and commercial assays (indicated as cmiR) or the miRquad in (a) tissues and (b) sera samples. [file 13046_2025_3590_MOESM3_ESM.tif]

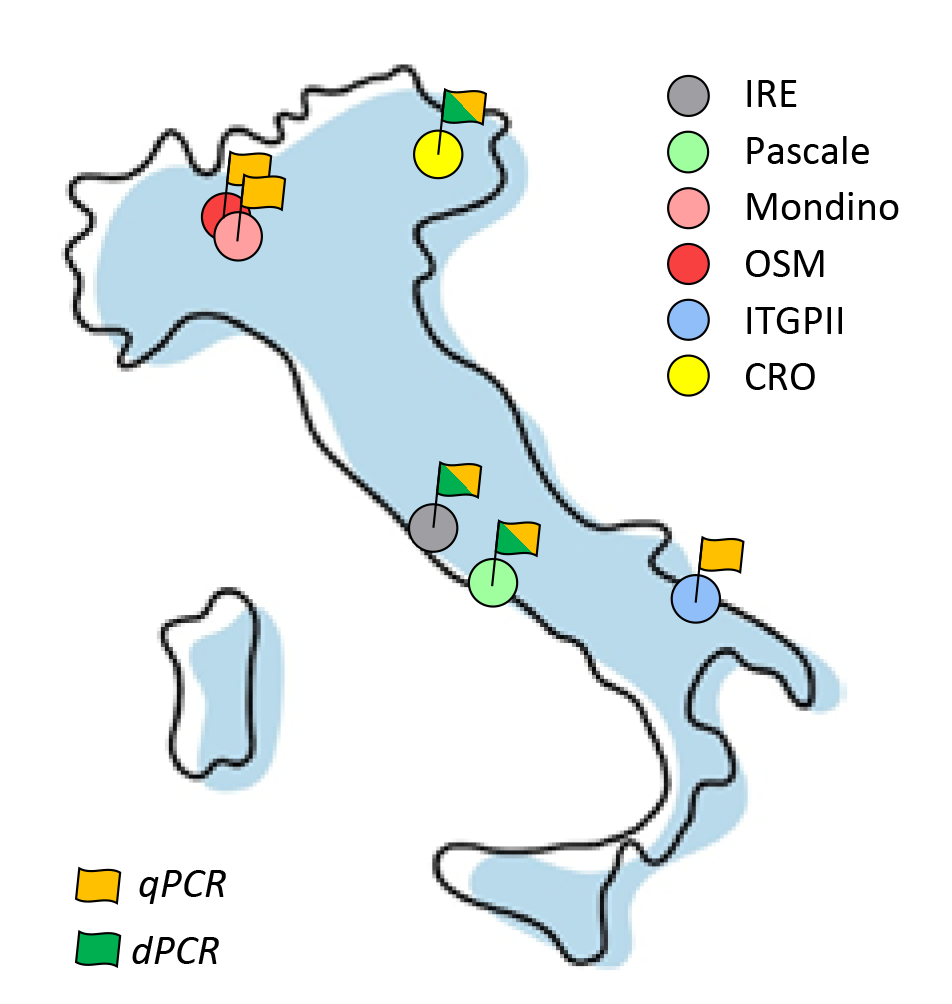

Supplement: Supplementary file 4 — Supplementary Material 4: Suppl. Fig. 1. Footprint of Centers participating in the ring study. Centers and platform availability (qPCR or dPCR) involved into the ring study are indicated. [file 13046_2025_3590_MOESM4_ESM.tif]
